# Parental burnout of parents of primary school students: an analysis from the perspective of job demands-resources

**DOI:** 10.3389/fpsyt.2023.1171489

**Published:** 2023-06-21

**Authors:** Jiangtao Zhao, Hua Hu, Siqin Zhao, Wenwen Li, Małgorzata Lipowska

**Affiliations:** ^1^School of Education, Zhengzhou University, Zhengzhou, China; ^2^School of Politics and Public Administration, Zhengzhou University, Zhengzhou, China; ^3^Faculty of Social Sciences, University of Gdańsk, Gdańsk, Poland; ^4^The No.3 Primary School of Jinshui Wenhua Road, Zhengzhou, China

**Keywords:** job demands-resources theory, parenting stress, perceived family support, resilience, parental burnout

## Abstract

**Objective:**

Based on the theory of Job Demands-Resources, this study has been set out to examine how parenting demands, parenting resources affect parental burnout of primary school students’ parents.

**Methods:**

An online survey with four scales (Parenting Stress Scale, Perceived Family Support Scale, Psychological Resilience Scale and Parental Burnout Scale) was completed by 600 parents of students from three primary schools in Central China. Structural equation models were implemented.

**Results:**

Parenting stress had a positive impact on parental burnout (*β* = 0.486, *p* < 0.001). Both perceived family support (*β* = −0.228, *p* < 0.001) and psychological resilience (*β* = −0.332, *p* = 0.001) had a negative impact on parental burnout. Perceived family support played a moderating role between parenting stress and parental burnout (*β* = −0.121, *p* < 0.001). Psychological resilience also played a moderating role between parenting stress and parental burnout (*β* = −0.201, *p <* 0.001). Psychological resilience partially mediated the relationship between perceived family support and parental burnout. The total effect was −0.290, with 95% CI (−0.350, −0.234). Direct effect was −0.228, with 95% CI (−0.283, −0.174), and indirect effect was −0.062, with 95% CI (−0.092, −0.037).

**Conclusion:**

Parental burnout may be reduced by increasing family support and self-improvement of psychological resilience. In the same way, the impact of parenting stress on parental burnout may be buffered under high-pressure situations.

## 1. Introduction

The concept of burnout was firstly mentioned in the literature of economics in the 1970s (1). Most of the related researches focused on certain occupations, and burnout were defined as a negative syndrome caused by long-term stress, manifested in physical and emotional fatigue, perfunctory work and the lack of a sense of accomplishment and efficacy at work (2). The Job Demands-Resources (JDR) theory is wildly used in the study of job burnout (3, 4). JD-R model proposing that working conditions can be categorized into two broad categories, job demands and job resources (5). Job demands refer to those physical, social, or organizational aspects of the job that require sustained physical and/or psychological (i.e., cognitive or emotional) effort on the part of the employee and are therefore associated with certain physiological and/or psychological costs (e.g., exhaustion) (6). They are typical predictors of fatigue and can positively predict job burnout (7). Job resources refer to those physical, psychological, social, or organizational aspects of the job that either/or: reduce job demands and the associated physiological and psychological costs; are functional in achieving work goals; stimulate personal growth, learning, and development (5). Job resources are predictors of engagement and can negatively predict job burnout (8). Excessive job demands and insufficient work resources affect the normal physical and psychological conditions of employees, resulting in job burnout and even health problems (9). Maintaining balance is the core of the theory: even if members of an organization face many job demands, but have sufficient resources to do so, the pressure they experience will be reduced, which may lower down the degree of job burnout (10).

Parenting is a complex and stressful job with many demands (11). It is suitable to apply the JD-R theory in the study of parental burnout. Parental burnout is typically characterized by parenting-related emotional exhaustion, emotional alienation from children and low efficacy with parental roles (10), which could be cumulative, negative and destructive (12). When the parenting resources needed to cope with the parenting stress are insufficient, which last for a long period of time, parents will be under greater pressures (13). Some parents with limited resources such as perceived family support and psychological resilience, may feel that they lack the energy to raise their children, just as they do not have enough resources to meet the demands of their jobs (14). If left untreated, parental burnout may cause some serious consequences for one’s marriage, work and the growth of children (15).

Parenting stress refers to the pressure that parents feel when they perform their parental roles within the parental subsystem. It is a subjective experience based on parenting requirements and it is affected by factors, such as parental personality traits, children’s behavior and parent–child interactions (16). When Previous researchers discussed job stress and job burnout, they regarded job burnout as a special form of job stress. However, due to in-depth research efforts, more scholars believe that the two are both related and different, and the difference is clearly reflected in the measurement dimension (17). Likewise, parenting stress and parental burnout differ significantly when measured: the tools used to measure parenting stress do not incorporate core dimensions of parental burnout, such as exhaustion and emotional distance from the child (18). Of course, the difference between stress and burnout is not only conceptual, research in the field of organization management has shown that job burnout is more damaging to individuals and organizations than job stress (19). Based on JD-R theory, many scholars have conducted a series of studies on various occupational groups. These researches have confirmed the negative effects caused by job burnout, indicating that the degree of job burnout will increase as the work stress increases (20, 21). Thus, we assume that the greater the parenting stress, the higher level of parental burnout.

Hobfoll divided resources into two categories: external resources and internal resources (22). Family support is a sub-dimension of social support (23), which is an external situational resource in the parenting process. Therefore, according to JD-R theory, this study suggests that family support may be an important factor in relieving parental burnout. The concept of social support is not unified in academia; it can be divided into two categories (24). One category is objective social support, which is support obtained in the social network. The second category is perceived social support, which is an individual’s subjective perception of social support, which aims to evaluate the individual’s access to the availability and adequacy of support (25). Studies have shown that compared with objective social support, perceived social support is more beneficial to the relief of psychological and emotional problems (26). Therefore, parental burnout from the perspective of perceived social support needs to be studied. The main effect model of social support on mental health indicates that social support can increase positive impact and reduce negative impact, and higher social support can directly lead to higher life satisfaction and lower job burnout (27). Whether an individual is under pressure or not, social support can maintain a good emotional experience and is beneficial to mental health (28). As a subsystem of society, perceived family support is the adaptation to the material, emotional and other resources provided by the family. The more family support is fully perceived, the lower the level of parental burnout; therefore, perceived family support can negatively predict parental burnout.

Psychological resilience refers to people’s ability to rebound or recover in difficult or stressful situations. It has certain emotional control functions and is categorized into the internal resources of individuals. The higher the individual’s level of psychological resilience, the more active to solve problems. Previous studies have shown that social support has a positive effect on psychological resilience (29). It can be speculated that perceived family support, as an external situational resource, can positively predict psychological resilience. Meanwhile, according to JD-R theory, psychological resilience, as an individual’s internal resource, can reduce burnout. From the above two aspects of psychological resilience, it can be seen that the higher the perceived family support, the more emotional balance can be maintained in the process of parenting, which is conducive to the increase of psychological resilience. As parenting resources, both can reduce parental burnout. Therefore, psychological resilience may play a mediating role in perceived family support and parental burnout.

Parenting stress and parenting resources (perceived family support, psychological resilience) not only directly affect parental burnout, they also interact. According to JD-R theory, parenting resources can reduce the impact of parenting stress on parental burnout, and the buffering effect of social support also verifies this view. Therefore, perceived family support plays a moderating role between parenting stress and parental burnout. In previous studies, psychological resilience has also primarily acted as a protective factor to buffer the effects of stress, thus explaining why some people can thrive in difficult situations (30). The moderating effect of psychological resilience has been well verified in groups, such as teachers and company employees (3, 4). However, the stress vulnerability hypothesis states that, in high-pressure situations, positive factors tend to decrease significantly or even lose their positive effects (31). Therefore, in the effect of parenting stress on parental burnout, whether perceived family support and psychological resilience are stress buffer factors or stress vulnerability factors needs to be further confirmed.

**Figure 1:**
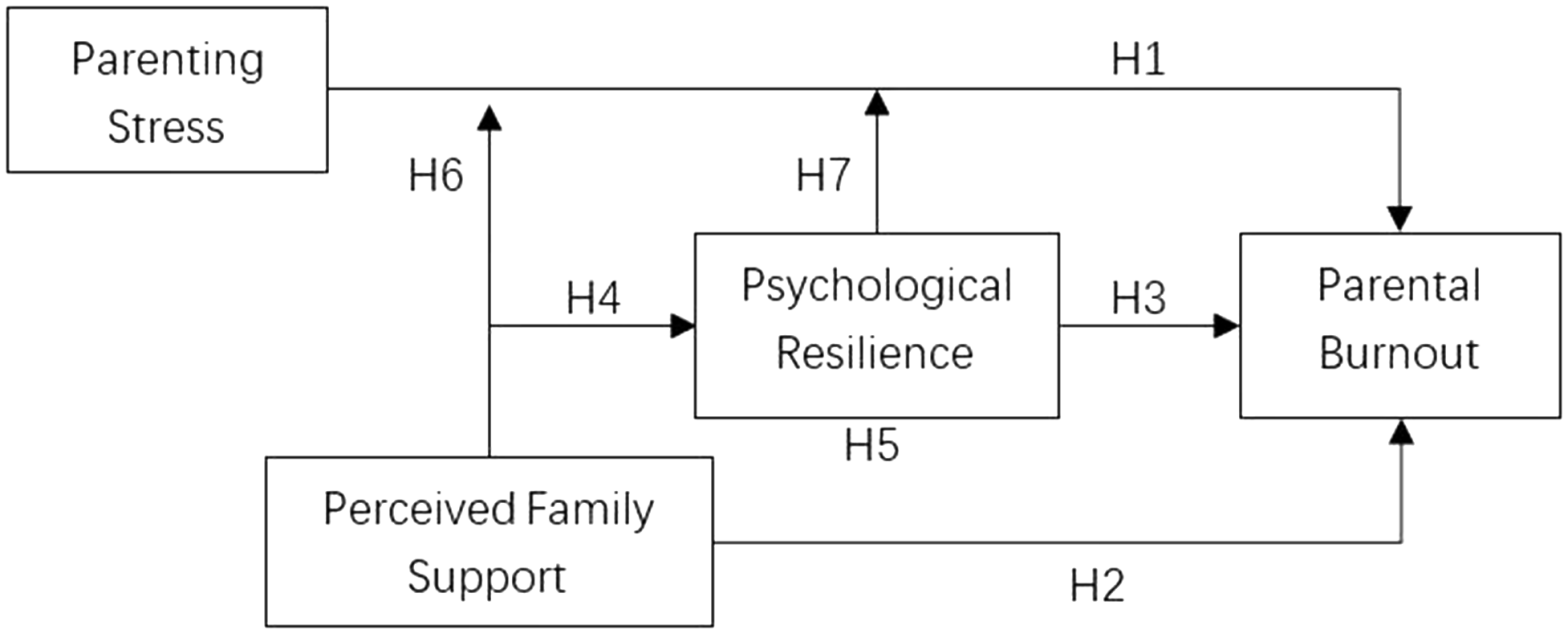
The hypothesized model.

Today, parents’ expectations for children are constantly increasing, making it more challenging for them to rear their children; thus, the pressure on parenting is increasing (32). Primary schooling is a critical period in children’s psychological development (33). With the implementation of the burden reduction policy, home-school cooperation has gradually increased, requiring parents to devote more time and energy to participating in their child’s upbringing. However, due to the difficulty in coordinating work and life, there is also an increased likelihood of burnout. Therefore, it is crucial to study the factors and mechanism of parental burnout of primary school students’ parents for a better understanding of parental burnout and the development of effective interventions. A model of the inter-relationships that need to be considered when examining parental burnout is proposed which details the interplay among factors such as parenting stress, perceived family support, psychological resilience and parental burnout. Based on the previous researches and the research aims, seven interrelated hypotheses are identified, as shown in Figure 1.

*H1*: Parenting stress may positively predict parental burnout.

*H2*: Perceived family support may negatively predict parental burnout.

*H3*: Psychological resilience may negatively predict parental burnout.

*H4*: Perceived family support may positively predict psychological resilience.

*H5*: Perceived family support can indirectly predict parental burnout through the mediating effect of psychological resilience.

*H6*: Perceived family support moderates the relationship between parenting stress and parental burnout.

*H7*: Psychological resilience moderates the relationship between parenting stress and parental burnout.

In terms of parenting requirements, the moderating effect of perceived family support and psychological resilience on parenting stress and parental burnout was explored. In terms of parenting resources, the psychological resilience of personal resources was introduced to explore the mechanism of perceived family support on parental burnout. The discussion of the above issues expands the research on parental burnout at the theoretical level; it also has important practical significance for how to reduce the parental burnout of primary school students’ parents.

## 2. Methods

### 2.1. Procedures and sample

A cluster sampling method was used, and 642 parents of three primary school students in Central China were selected, a link of the online survey Questionnaire with four scales (Parenting Stress Scale, Perceived Family Support Scale, Psychological Resilience Scale, and Parental Burnout Scale) was distributed to head teachers, who then sent the link to the selected parents. Questionnaires with short completion time were deleted, and 600 valid data were collected with a valid response rate of 93.5%.

### 2.2. Research tools

#### 2.2.1. Simplified parenting stress scale

The Simplified Parenting Stress Scale, compiled by Abidin and revised by Ren (34), has 36 items in three dimensions: parenting distress, parent–child interaction disorder and difficult children. The item choices range from “strongly disagree” to “strongly agree,” each ranked on a scale of 1–5 points. The higher the score, the greater the parenting stress. In our study, the Cronbach’s α of the total scale was 0.947, and the Cronbach’s α of the three dimensions were 0.901, 0.896, and 0.892, respectively.

#### 2.2.2. Multidimensional perceived social support scale

This scale was compiled by Zimet et al. (23); it has a total of 12 items, including three dimensions of family support, friend support and significant other support. The study used four items from the family support dimension and the options ranged from “strongly disagree” to “strongly agree,” each ranked on a scale of 1–7 points. The higher the score, the stronger the perceived family support. The Cronbach’s α in our study was 0.898.

#### 2.2.3. Simplified psychological resilience scale

Connor et al. developed the Campbell Sills Simplified One-dimensional Psychological Resilience Scale (35); it contains a total of 10 questions, with options ranging from “never” to “almost always,” each ranked on a scale of 0–4 points. The higher the score, the stronger the psychological resilience. The Cronbach’s α in our study was 0.896.

#### 2.2.4. Simplified parental burnout scale

This scale was compiled by Cheng et al. (36) found that the scale was one-dimensional when he conducted a test of Chinese localization. Wang et al. (37) research results confirmed Cheng’s point of view and revised the simplified version. The present study adopted the simplified parental burnout scale revised by Wang Wei, with a total of 7 items. The options range from “never” to “every day,” and each is ranked on a scale of 1–7 points, with higher scores indicating higher levels of parental burnout. The Cronbach’s α in our study was 0.925.

### 2.3. Data analysis

IBM SPSS25.0 and Process plug-in were used for data analysis. The correlation analysis, mediation effect and moderating effect test were conducted. The Cronbach’s α was used for internal consistency reliability of measurements. The correlation between parenting stress, perceived family support, psychological resilience and parental burnout was tested by Pearson correlation analysis. Hierarchical regression was used to test the moderating effect. To further reveal the essence of the moderating effects of perceived family support and psychological resilience, the study used the simple slope test to analyze the effect of parenting stress on parental burnout when perceived family support and psychological resilience were one standard deviation from the average score (38). The mediating effect test used model 4 in Process, and bootstrapping (5,000 times) was used to provide confidence intervals. The Harman univariate test was used for the common method bias (39). The results showed that there were 9 common factors with eigenvalues greater than 1. The first common factor explained 30.88% of the total variance, which was less than the critical value of 40%, which showed that there was no significant common method bias.

## 3. Findings

### 3.1. Demographics

A detailed demographic profile is presented in Table 1. A total of 181 participants were males (30.2%) and 419 were females (69.8%). Of the respondents, 19 (3.2%) were 30 years of age or younger, 314 (52.3%) were in the 31–35 age range, 209 (34.8%) were in the 36–40 age range and 58 (9.7%) were 41 years of age or older. In terms of the number of children 67 (11.2%) respondents have one child, and 533 (88.8%) have two or more children.

**Table 1:** Demographic characteristics of the sample (*n* = 600).

Variables	Categories	*n*	%
Gender	Male	419	69.8
	Female	181	30.2
Age (y)	≤30	19	3.2
	31–35	314	52.3
	36–40	209	34.8
	≥41	58	9.7
Number of children	1	67	11.2
	≥2	533	88.8

### 3.2. Descriptive statistics and correlation analysis

As seen in Table 2, parenting stress was significantly negatively correlated with perceived family support and psychological resilience, and it was significantly positively correlated with parental burnout. Perceived family support was significantly positively correlated with psychological resilience, and significantly negatively correlated with parental burnout. Psychological resilience was significantly negatively correlated with parental burnout.

**Table 2:** Descriptive statistics and correlation analysis (*n* = 600).

	*^−^x(s)*	1	2	3	4
1. Parenting stress	2.475 (0.591)	1			
2. Perceived family support	4.961 (1.341)	−0.509***	1		
3. Psychological resilience	3.702 (0.622)	−0.497***	0.411***	1	
4. Parental burnout	1.635 (0.939)	0.496***	−0.432***	−0.362***	1

*p < 0.05, **p < 0.01, ***p < 0.001, the following are the same.

### 3.3. The effect of parenting stress on parental burnout: the moderating role of perceived family support and psychological resilience

After centralizing the variables, hierarchical regression was used to further explore the moderating role of family support and psychological resilience on parenting stress and parental burnout. First, the control variables, gender, age and the number of children raised, were entered into regression (M 1). Second, the independent variable, parenting stress, was entered into regression (M 2). Third, the moderator variables, perceived family support (M 3) and psychological resilience (M 5), were entered into the regression. Finally, the interaction terms, parenting stress × perceived family support (M 4), parenting stress × resilience (M 6), were entered into the regression. The regression results are shown in Table 3.

**Table 3:** Hierarchical regression results for parental burnout.

Variable		Parental burnout				
	M 1	M 2	M 3	M 4	M 5	M 6
Gender	0.198***	0.144***	0.122***	0.131***	0.138***	0.127***
Age	0.033	0.028	0.009	0.010	0.019	0.016
Number of children	0.009	−0.047	−0.044	−0.045	−0.050	−0.048
Parenting stress		0.486***	0.372***	0.379***	0.413***	0.440***
Perceived family support			−0.227***	−0.206***		
Psychological resilience					−0.148***	−0.139***
Parenting stress × perceived family support				−0.121***		
Parenting Stress × psychological resilience						−0.201***
*R* ^2^	0.039	0.268	0.306	0.320	0.285	0.325
*Adj. R* ^2^	0.034	0.264	0.300	0.313	0.279	0.318
△*R*^2^	0.039	0.230	0.038	0.014	0.016	0.040
△*F*	7.960	186.987***	32.098***	12.448***	13.648***	34.872***

***p < 0.001.

M 1 only tested the control variables, in which gender had a significant impact on parental burnout, while age and number of children had no significant impact on parental burnout. In M 2, due to the addition of parenting stress, the adjusted R^2^ was 0.264, increasing by 0.230. Parenting stress had a positive predictive effect on parental burnout (*β* = 0.486, *p* < 0.001), H1 was supported. In M3 and M5, due to the addition of perceived family support and psychological resilience, the adjusted R^2^ were 0.300 and 0.279, increasing by 0.038 and 0.016, respectively. Both perceived family support (*β* = −0.227, *p* < 0.001) and psychological resilience (*β* = −0.148, *p* < 0.001) had negative effects on the parental burnout. In M4 and M6, due to the addition of two interaction items. The adjusted R^2^ were 0.313 and 0.318, increasing by 0.014 and 0.040, respectively. The interaction item of parenting stress and perceived family support (*β* = −0.121, *p* < 0.001) and the interaction item of parenting stress and resilience (*β* = −0.201, *p* < 0.001), were both negative to predict parental burnout. In conclusion, both perceived family support and psychological resilience moderate the relationship between parenting stress and parental burnout, H6 and H7 were supported.

**Figure 2:**
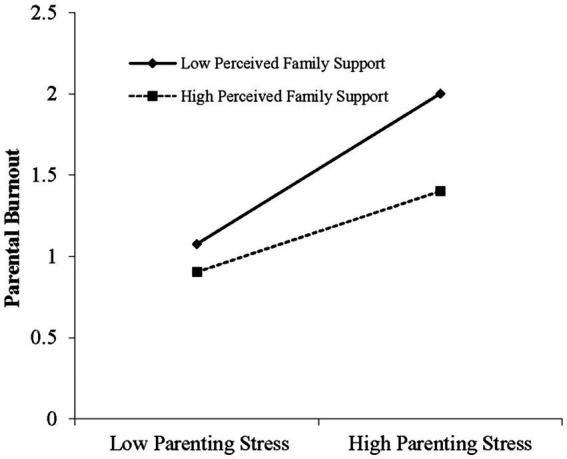
The moderating effect of perceived family support on the relationship between parenting stress and parental burnout.

To further reveal the essence of the moderating effects of perceived family support. As seen in Figure 2, when the level of perceived family support was low (M−1SD), parenting stress had a significant positive predictive effect on parental burnout (simple slope = 0.784, *t* = 10.296, *p* < 0.001). When the level of perceived family support was high (M + 1SD), the positive predictive effect of parenting stress on parental burnout was also significant, but the predictive effect was significantly weakened (simple slope = 0.422, *t* = 5.541, *p* < 0.001).

**Figure 3:**
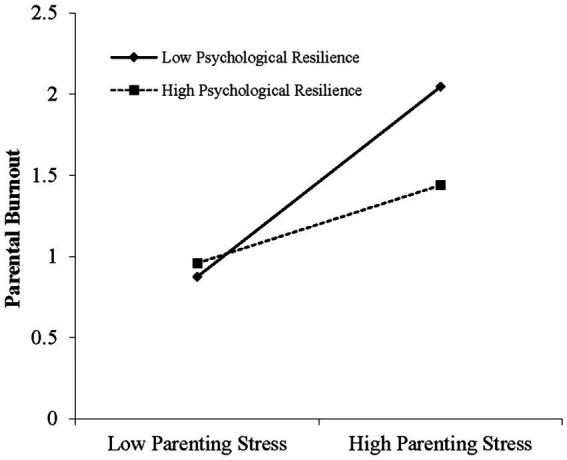
The moderating effect of psychological resilience on the relationship between parenting stress and parental burnout.

To further reveal the essence of the moderating effects of psychological resilience. As seen in Figure 3, when the level of psychological resilience was low (M−1SD), parenting stress had a significant positive predictive effect on parental burnout (simple slope = 0.991, *t* = 11.397, *p* < 0.001). When the level of psychological resilience was high (M + 1SD), the positive predictive effect of parenting stress on parental burnout was also significant, and the predictive effect was also significantly weakened (simple slope = 0.407, *t* = 5.707, *p* < 0.001).

### 3.4. The effect of perceived family support on parental burnout: the mediating role of psychological resilience

After controlling for gender, age and the number of children, the mediating effect of psychological resilience between perceived family support and parental burnout was tested. The results were shown in Tables 4, 5.

**Table 4:** Mediation model test of psychological resilience.

Regression equation		Overall fit coefficient		Regression coefficient significance	
Outcome variable	Predictor variable	*R^2^*	*F*	*β*	*t*
Parental burnout		0.205	38.337***		
	Gender			0.277	3.635***
	Age			−0.004	−0.082
	Number of children			−0.027	−0.252
	Perceived family support			−0.290	−11.159***
Psychological resilience		0.174	31.365***		
	Gender			−0.044	−0.856
	Age			−0.026	−0.833
	Number of children			−0.119	−1.619
	Perceived family support			0.186	10.629***
Parental burnout		0.245	38.526***		
	Gender			0.262	3.528**
	Age			−0.012	−0.275
	Number of children			−0.067	−0.629
	Perceived family support			−0.228	−8.249***
	Psychological resilience			−0.332	−5.607***

**p < 0.01, ***p < 0.001.

**Table 5:** Decomposition table of total effect, direct effect, and mediating effect.

	Effect	BootSE	BootLLCI	BootULCI	Effect ratio
Psychological resilience	−0.062	0.014	−0.092	−0.037	21.38%
Direct effect	−0.228	0.028	−0.283	−0.174	78.62%
Total effect	−0.290	0.030	−0.350	−0.234	

Table 4 shows that perceived family support had a negative effect on the parental burnout (*β* = −0.290, *p* < 0.001), and a positive effect on the psychological resilience (*β* = 0.186, *p* < 0.001), H2 and H4 were supported. When both perceived family support and psychological resilience were included in the model, the direct effect of perceived family support on parental burnout was significant (*β* = −0.228, *p* < 0.001), and psychological resilience was also significant (*β* = −0.332, *p* < 0.001), H3 was also supported.

Table 5 shows that the bootstrap 95% confidence intervals of the indirect of psychological resilience (cl = −0.092; −0.037) and the direct effect of perceived family support (cl = −0.283; −0.174) on parental burnout of primary and middle school students did not contain 0; The direct effect (−0.228) and the mediation effect (−0.062) accounted for 78.62 and 21.38% of the total effect. Thus, psychological resilience played a partial mediating role between perceived family support and parental burnout. H5 was supported.

## 4. Discussion

Based on JD-R theory, the present study investigated the parenting from the perspectives of risk (parenting stress) and protection (parenting resources) to explore the influencing factors of parental burnout and its mechanisms. It expands the application of this theory in parenting setting. The results showed that parenting stress and parental burnout were positively correlated, from which we can see that parenting stress is a risk factor. Perceived family support, which is external situational parenting resources, and psychological resilience, which is internal individual parenting resources, were negatively correlated with parental burnout respectively, which can be taken as two protective factors. The research findings are in accordance with previous studies on workers, teachers, doctors and other occupations (40–42). However, compared with the average score of burnout, the parental burnout was significantly lower than job burnout. This may be because work is optional for most people, but children are not. For most parents, raising children is the most important thing in their life, they have a high tolerance in the process, even if the children give them a lot of headaches, they will find a way to overcome.

JD-R theory initially defined job resources as external situational resources brought by the work environment (5). When investigating burnout, few studies have examined internal individual resources. After confirming that perceived family support can significantly and negatively predict the parental burnout of the parents of primary school students, this study introduced psychological resilience as an individual resource to further explore its mediating role. The results suggest that psychological resilience as an internal resource plays a partial mediating role between perceived family support and parental burnout. To certain extent, situational resources shape individual resources and reduce the generation of burnout; that is, parents with higher perceived family support tend to have higher psychological resilience. Therefore, we should improve the level of co-parenting, so as to improve psychological resilience. Individuals with high psychological resilience tend to be optimistic, tough and other characteristics (42), and could be more active in parenting, reducing the possibility of parental burnout.

The study also found that both perceived family support and psychological resilience play a moderating role between parenting stress and parental burnout. High perceived family support and psychological resilience could effectively reduce the impact of parenting stress on parental burnout, and the results support the stress buffer hypothesis. Today, working couples have become the mainstream in society. Although this relieves some of the economic pressure, the high cost of parenting is still a problem for most families. At the same time, due to external work pressure, parents will have less energy for parenting, so more family support can make up for the lack of resources in raising children and help create a harmonious family atmosphere. Furthermore, related studies have demonstrated that psychological resilience may change over time and it can be learned (43). For parents of primary school students who are experiencing parental burnout, some skills training related to improving their psychological resilience, such as cognitive reconstruction and optimism, may prevent or reduce the impact of parenting stress on parental burnout.

## 5. Limitations

Since the survey used in this study collected cross-sectional data, it is difficult to fully prove the causal relationship between variables. In the future, we can further explore the problem of parental burnout by conducting longitudinal research. Moreover, the variable, stress, can be categorized as positive challenging stress and negative obstructive stress, which may have different effects on burnout. As an external situational resource, the moderating role of family support and internal personal resource resilience needs to be further explored as well.

## 6. Conclusion

In summary, there is a significant positive correlation between parenting stress and parental burnout in the parents of primary school students. Perceived family support, psychological resilience and parental burnout were significantly negatively correlated. Psychological resilience partially mediates the relationship between perceived family support and parental burnout. Both perceived family support and psychological resilience moderate parenting stress and parental burnout.

## Data availability statement

The raw data supporting the conclusions of this article will be made available by the authors, without undue reservation.

## Ethics statement

All procedures involving human participants in this study were in accordance with the ethical standards of the Zhengzhou University. The patients/participants provided their written informed consent to participate in this study.

## Author contributions

JZ was responsible for research design and framework and with HH participated in data acquisition and analysis, drafting, and revising the manuscript. SZ, WL, and ML carefully revised the manuscript to maintain accuracy. All authors reviewed and approved the final version of the manuscript.

## Funding

This work was supported by the National Social Science Fund of China (grant no. BIA180208) and Henan Provincial Science and Technology Commission (grant no. HNGD2022022).

## Conflict of interest

The authors declare that the research was conducted in the absence of any commercial or financial relationships that could be construed as a potential conflict of interest.

## Publisher’s note

All claims expressed in this article are solely those of the authors and do not necessarily represent those of their affiliated organizations, or those of the publisher, the editors and the reviewers. Any product that may be evaluated in this article, or claim that may be made by its manufacturer, is not guaranteed or endorsed by the publisher.
